# CHA_2_DS_2_-VASc score, cerebral small vessel disease, and frailty in older patients with atrial fibrillation

**DOI:** 10.1038/s41598-020-75256-6

**Published:** 2020-10-30

**Authors:** Jung-Yeon Choi, Leonard Sunwoo, Sun-wook Kim, Kwang-il Kim, Cheol-Ho Kim

**Affiliations:** 1 Department of Internal Medicine, Seoul National University Bundang Hospital, 82, Gumi-ro 173beon-gil, Seongnam-si, Kyeongi-do 13620 Republic of Korea; 2 Seoul National University College of Medicine, 103 Daehak-ro, Jongno-gu, Seoul 03080 Republic of Korea; 3 Department of Radiology, Seoul National University Bundang Hospital, 82, Gumi-ro 173beon-gil, Seongnam-si, Kyeongi-do 13620 Republic of Korea

**Keywords:** Neuroscience, Cardiology, Risk factors

## Abstract

The CHA_2_DS_2_-VASc score is a validated predictor of ischemic stroke in atrial fibrillation (AF) patients. However, data are limited on whether the CHA_2_DS_2_-VASc score is associated with subclinical brain structural changes or physical frailty among older AF patients. We assessed the relationship between CHA_2_DS_2_-VASc scores and brain structural changes or physical frailty in AF patients without history of stroke. Overall, 117 patients completed a comprehensive geriatric assessment for physical frailty. In brain magnetic resonance imaging sub-study (n = 49), brain volume and white matter hyperintensity lesion burden were automatically quantified using the LESIONQUANT software program. Patients with high risk of CHA_2_DS_2_-VASc scores (≥ 2 in men or ≥ 3 in women) tended to be older and had more comorbidities, higher frailty index, and slower gait speed. Total white matter hyperintensity lesion burden was higher in those with high risk of CHA_2_DS_2_-VASc score than in those with intermediate risk (score of 1 in men or 2 in women) of CHA_2_DS_2_-VASc score (1.67 [interquartile range: 0.70–3.45] vs. 0.64 [0.19–1.44], *p* = 0.036). Cognitive function was associated with brain volume, but gait speed was related with white matter hyperintensity lesion burden. In conclusion, we showed a positive relationship between CHA_2_DS_2_-VASc scores, white matter hyperintensity lesion burden, and physical frailty in older AF patients. Subclinical brain changes associated with high CHA_2_DS_2_-VASc scores may predict physical frailty risk.

## Introduction

Atrial fibrillation (AF) is one of the common sustained arrhythmias among older adults and is known to be associated with increased risk of mortality, stroke, thromboembolism, or heart failure^1^. The estimated prevalence of AF has been reported to range from 0.4 to 2%, world widely^2^. In Korea, prevalence of AF progressively increased from 0.73% in 2006 to 1.53% in 2015. Especially, annual AF incidence in patients aged ≥ 80 years increased significantly while the incidences in all other age groups decreased^3^. AF significantly influences the quality of life of older adults because the prevalence and attribution risk of stroke associated with AF significantly increase with aging^4^. Therefore, risk stratification for subsequent stroke or thromboembolism, treatment-related complications, and further functional decline in older population is important to determine individualised preventive or treatment strategy and goal of care. Among the risk stratification tools, the CHA_2_DS_2_-VASc (Congestive Heart Failure, Hypertension, Age ≥ 75 (doubled), Diabetes Mellitus, Prior Stroke or Transient Ischemic Attack (doubled), Vascular Disease, Age 65–74, Female) score can predict heart failure, cardiovascular hospitalisation, and death, and is thus widely used to identify the future risk of ischemic stroke^5–9^.

Regarding subclinical brain changes, cerebral small vessel disease (SVD) is a common finding in older adults. The reported prevalence of SVD in older population varies greatly across different studies, ranging from 8 to 33% for lacunes^10^, 3 to 34% for cerebral microbleeds^11,12^, and 39 to 96% for white matter hyperinstensitiy (WMH)^13^. The burden of white matter hyperintensity (WMH) lesions can be objectively evaluated with the automated volumetric method, and the results are in concordance with those obtained using classical validated visual rating scales^14^. Although SVD is a major contributor to dementia, the relationship between AF and the CHA_2_DS_2_-VASc score and physical or cognitive function remains inconclusive, especially in patients without stroke history^15,16^.

A state of decreased physiologic reserve and increased vulnerability is known to be associated with increased risk of adverse health-related outcomes^17^. The comprehensive geriatric assessment (CGA) is widely used with a multidimensional interdisciplinary diagnostic approach for determining frail older adults’ medical, psychological, and functional capacity^18^. In our previous study, we identified that the CHA_2_DS_2_-VASc score is positively associated with frailty status and that frailty assessment can be used to predict mortality in older AF patients and provides additional prognostic value, along with the CHA_2_DS_2_-VASc and HAS-BLED scores. However, studies on the underlying mechanism linking frailty and the CHA_2_DS_2_-VASc score in AF patients are limited.

Brain WMH lesions lead to cognitive decline and are correlated with frailty in older adults^19,20^. Patients with higher CHA_2_DS_2_-VASc score are considered to have a higher burden of subclinical WMH lesions, which may affect physical frailty in older AF patients. Therefore, we aimed to evaluate the relationship between CHA_2_DS_2_-VASc scores, brain WMH lesions, and frailty. To obtain evidence for the mechanisms underlying the higher incidence of physical frailty in the high risk of CHA_2_DS_2_-VASc score group, the correlation between CHA_2_DS_2_-VASc scores, subclinical brain structural parameter (volume or WMH lesion), and frailty was evaluated in a sub-study of patients without stroke or transient ischemic attack who underwent brain MRI.

## Results

### Participants’ characteristics

Of the 133 participants recruited, 117 were included in the analysis. The brain MRI sub-study comprised 49 patients. The clinicodemographic characteristics according to the CHA_2_DS_2_-VASc score are presented in Table 1. The median age was 78 years (interquartile range [IQR]; 74–82), and 45.3% (n = 53) were female. Among the participants, 104 (88.9%) were persistent and 13 (11.1%) were paroxysmal AF. Comorbidities were prevalent among the participants (hypertension, 64.1%; diabetes, 27.4%; and congestive heart failure, 8.5%). Moreover, 57 patients (48.7%) were treated with antiplatelet agents and 65 (55.6%) with anticoagulants, and 2 (1.7%) did not take any antithrombotic agent. Polypharmacy was common in the study population, that is, 86 (73.5%) and 31 patients (26.5%) were respectively taking > 5 and > 10 drugs simultaneously. Results of the CGA are described in Table 1.

**Table 1:** Clinical characteristics and comprehensive geriatric assessment according to the risk of CHA2DS2-VASc score.

Variables	Total (N = 117)	Intermediate risk of CHA_2_DS_2_-VASc (n = 13)	High risk of CHA_2_DS_2_-VASc^†^ (n = 104)	*p* value
**Demographics**				
Age (year)	78 (74–82)	72 (70.5–74)	78.5 (76–83)	*** < 0.001***
Sex, female (%)	53 (45.3%)	6 (46.2%)	47 (45.2%)	0.948
BMI, kg/m^2^	25.2 (23.0–27.1)	25.2 (22.2–27.4)	25.1 (23.0–27.0)	0.752
**Medication and risk stratification**				
Antiplatelet use	57 (48.7%)	9 (69.2%)	48 (46.2%)	0.147
Anticoagulation use	65 (55.6%)	4 (30.8%)	61 (58.7%)	0.076
Elevated HAS-BLED risk (≥ 3)	29 (24.8%)	1 (7.7%)	28 (26.9%)	0.181
**Comprehensive geriatric assessment**				
CCI	1 (0–2)	0 (0–1.5)	1 (0–2)	***0.048***
ADL dependency	13 (11.1%)	0 (0%)	13 (12.5%)	NA
IADL dependency	21 (17.9%)	0 (0%)	21 (20.2%)	NA
TUGT^‡^	13 (10–14)	10 (9–12)	13 (11–14.75)	***0.004***
Gait speed^§^	0.98 (0.79–1.17)	1.23 (0.96–1.41)	0.97 (0.75–1.14)	***0.007***
Grip strength	24.5 (18.9–30.8)	30.1 (22.1–39.6)	24.1 (18.4–30.5)	0.120
MMSE-KC	26 (22–28)	28 (25.5–28)	26 (22–28)	0.161
SGDS-K^¶^	3 (1–7)	2 (0–2.5)	3 (1–7.5)	***0.025***
MNA	26 (23–27)	28 (26.3–28)	25.5 (22.5–27)	***0.001***
Polypharmacy (≥ 5 drugs)	86 (73.5%)	4 (30.8%)	82 (48.8%)	***0.001***
Frailty index	0.06 (0–0.17)	0 (0–0.06)	0.06 (0–0.19)	***0.007***

Data are presented as median (25–75th percentiles) or number of participants (percentages).

^†^High risk of CHA_2_DS_2_-VASc score refers to score ≥ 2 in men or ≥ 3 in women and intermediate risk of CHA_2_DS_2_-VASc score refers to score 1 in men or score 2 in women.

^‡^Data were missing for 12 patients.

^§^Data were missing for seven patients.

^¶^Data were missing for three patients.

ADL, activity of daily living; BMI, body mass index; CHA_2_DS_2_-VASc, congestive heart failure, hypertension, age ≥ 75 (doubled), diabetes mellitus, prior stroke or transient ischemic attack (doubled), vascular disease, age 65–74, female; CCI, Charlson Comorbidity Index; HAS-BLED, Hypertension, Abnormal renal/liver function, Stroke, Bleeding history or predisposition, Labile INR, Elderly, Drugs/alcohol concomitantly; IADL, instrumental activity of daily living; MMSE-KC, Korean version of Mini–Mental State Examination; MNA, Mini Nutritional Assessment; SGDS-K, short form of the Korean Geriatric Depression Scale; TUGT, timed up and go test.

The median CHA_2_DS_2_-VASc score and HAS-BLED score were 4 (interquartile range [IQR] 3–5) and 2 (IQR 2–2.5), respectively. In total, 104 (88.9%) patients had a high risk of CHA_2_DS_2_-VASc score. Patients who had high risk of CHA_2_DS_2_-VASc score tended to be older and taking > 5 medications; they also had more comorbidities, higher frailty index, and slower gait speed and timed up and go test (TUGT) time (Table 1).

We analysed brain variables (volume of brain structures and WMH lesion burden and anatomical distribution) according to the CHA_2_DS_2_-VASc score groups. With regard to the brain structure volume, only the hippocampus volumes were larger in the intermediate risk of CHA_2_DS_2_-VASc score group than in the high risk of CHA_2_DS_2_-VASc score group. After adjustment for the intracranial volume (ICV), all brain structural volumes were not statistically different between the groups. The total WMH lesion volume adjusted with the ICV and lesion burden were significantly higher in the high risk of CHA_2_DS_2_-VASc score group (Supplementary Fig. 1). With respect to the anatomical distribution of the WMH lesions, the area of the deep white matter WMH lesion was especially larger in the high risk of CHA_2_DS_2_-VASc score group (Table 2).

**Table 2:** Brain MRI variables according to the risk of CHA_2_DS_2_-VASc score.

Brain structure	Intermediate risk of CHA_2_DS_2_-VASc (n = 9)		High risk of CHA_2_DS_2_-VASc (n = 40)		*p* value	
	Volume (cm^3^)	% ICV (%)	Volume (cm^3^)	% ICV (%)	Volume	%ICV
Whole brain	1043.6 (980.8–1154.2)	69.7 (68.4–72.7)	986.0 (918.2–1052.3)	68.9 (67.2–71.5)	0.064	0.408
Superior lateral ventricles	46.9 (28.0–74.8)	3.19 (2.11–4.27)	45.5 (31.3–60.1)	3.03 (2.24–4.19)	0.949	0.970
Thalamus	13.3 (12.3–15.6)	0.89 (0.88–0.95)	12.9 (12.2–13.7)	0.91 (0.85–0.97)	0.313	0.889
Cortical gray matter	458.1 (436.0–499.0)	30.8 (29.5–31.4)	433.1 (404.0–452.8)	30.2 (28.7–31.6)	0.053	0.770
Cerebral white matter	412.8 (358.8–455.1)	27.1 (26.3–28.2)	380.1 (338.2–404.1)	26.0 (25.1–27.7)	0.053	0.107
3rd ventricle	2.71 (1.95–3.60)	0.18 (0.14–0.21)	2.41 (2.12–2.84)	0.17 (0.15–0.20)	0.694	0.751
Hippocampi	6.69 (6.15–6.88)	0.44 (0.42–0.48)	5.93 (5.63–6.44)	0.43 (0.38–0.45)	***0.002***	0.140
Inferior lateral ventricles	3.09 (2.72–3.87)	0.22 (0.19–0.25)	3.79 (2.77–5.00)	0.26 (0.21–0.33)	0.408	0.091
**WMH lesion burden**						
Lesion count (n)	23.0 (6.5–32.5)		25.5 (15.0–50.75)		0.170	
Lesion volume (cm^3^)	3.26 (0.80–5.32)		6.04 (2.78–13.94)		***0.050 (0.0497)***	
% ICV (%)	0.17 (0.06–0.36)		0.44 (0.19–0.90)		***0.027***	
Lesion Burden	0.64 (0.19–1.44)		1.67 (0.70–3.45)		***0.036***	
**WMH lesion anatomical distribution (cm**^**3**^**)**						
Leukocortical	0.09 (0.01–0.27)		0.07 (0.01–0.39)		0.829	
Periventricular	2.87 (0.62–4.66)		4.13 (2.32–10.85)		0.077	
Deep white matter	0.12 (0.01–0.42)		0.48 (0.19–1.34)		***0.027***	

Data are presented as median (25–75th percentiles). High risk of CHA2DS2-VASc score refers to score ≥ 2 in men or ≥ 3 in women and intermediate risk of CHA2DS2-VASc score refers to score 1 in men or score 2 in women.

CHA_2_DS_2_-VASc: congestive heart failure, hypertension, age ≥ 75 (doubled), diabetes mellitus, prior stroke or transient ischemic attack (doubled), vascular disease, age 65–74, female; ICV, intracranial volume; MRI, magnetic resonance imaging; WMH, white matter hyperintensity.

### Relationship between brain image parameters and cognitive function or physical frailty

Compared with the lower mini-mental state examination (MMSE) score (≤ 24) group (n = 14), the higher MMSE score (> 24) group (n = 35) had larger whole brain, thalamus, cortical grey matter, cerebral white matter, and hippocampi volumes. After adjusting for the ICV, cerebral white matter volume was larger in the higher MMSE score group (*p* = 0.037). However, the WMH lesion burden or lesion anatomical distribution was not statistically different between the higher and lower MMSE score groups (Supplementary Table 1). Participants who had a faster gait speed (≥ 1.2 m/s, n = 12) had larger whole brain, cortical grey matter, cerebral white matter, and hippocampi volumes than did participants who had a slower gait speed (< 1.2 m/s, n = 36); however, this statistical significance disappeared when the result was adjusted for the ICV. The slower gait speed group had a larger WMH lesion volume (*p* = 0.012) and lesion volume adjusted for ICV (*p* = 0.006), and higher lesion burden (*p* = 0.006). For the WMH lesion anatomical distribution, the slower gait speed group had larger lesion volumes in the periventricular area (*p* = 0.011) (Supplementary Table 2).

## Discussion

**Figure 1:**
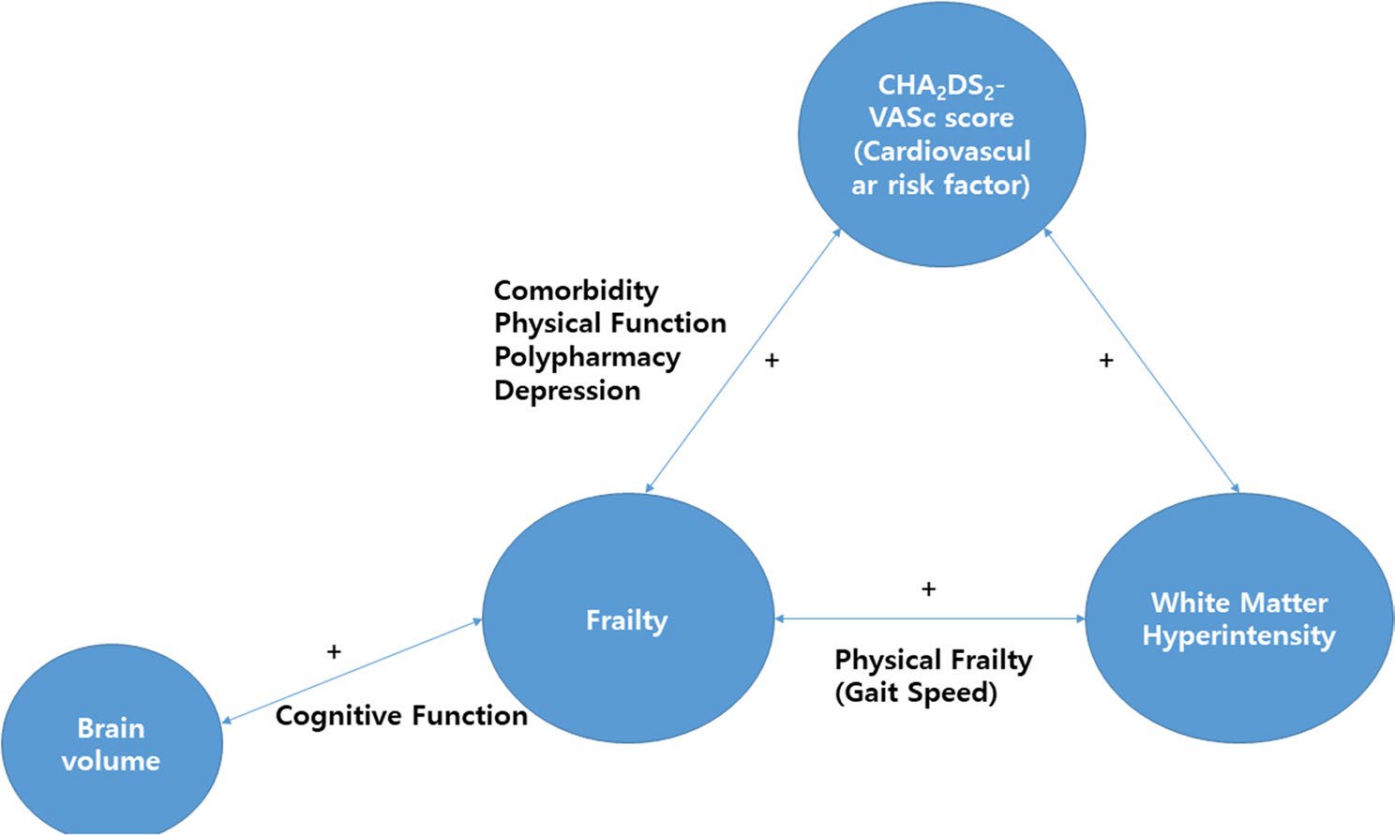
Relationship between the CHA_2_DS_2_-VASc score, brain white matter hyperintensity lesion, brain volume, and frailty. The CHA_2_DS_2_-VASc score, physical frailty, and white matter hyperintensity lesion/brain volume have a significant ( +) interrelationship.

Patients with high risk of CHA_2_DS_2_-VASc score tended to be more frail and slower and showed poor physical performance when compared with those with intermediate risk of CHA_2_DS_2_-VASc score. Further, the intermediate risk of CHA_2_DS_2_-VASc score group had a lower cerebral SVD burden. Slow gait speed was also correlated with higher WMH lesion burden, and cognitive function was associated with brain volumes. Regarding the lesion anatomical distribution, the WMH lesion volume was significantly larger in the deep white matter among those with high risk of CHA_2_DS_2_-VASc score. A higher WMH lesion burden in the periventricular area was associated with slow gait speed (Fig. 1).

**Figure 2:**
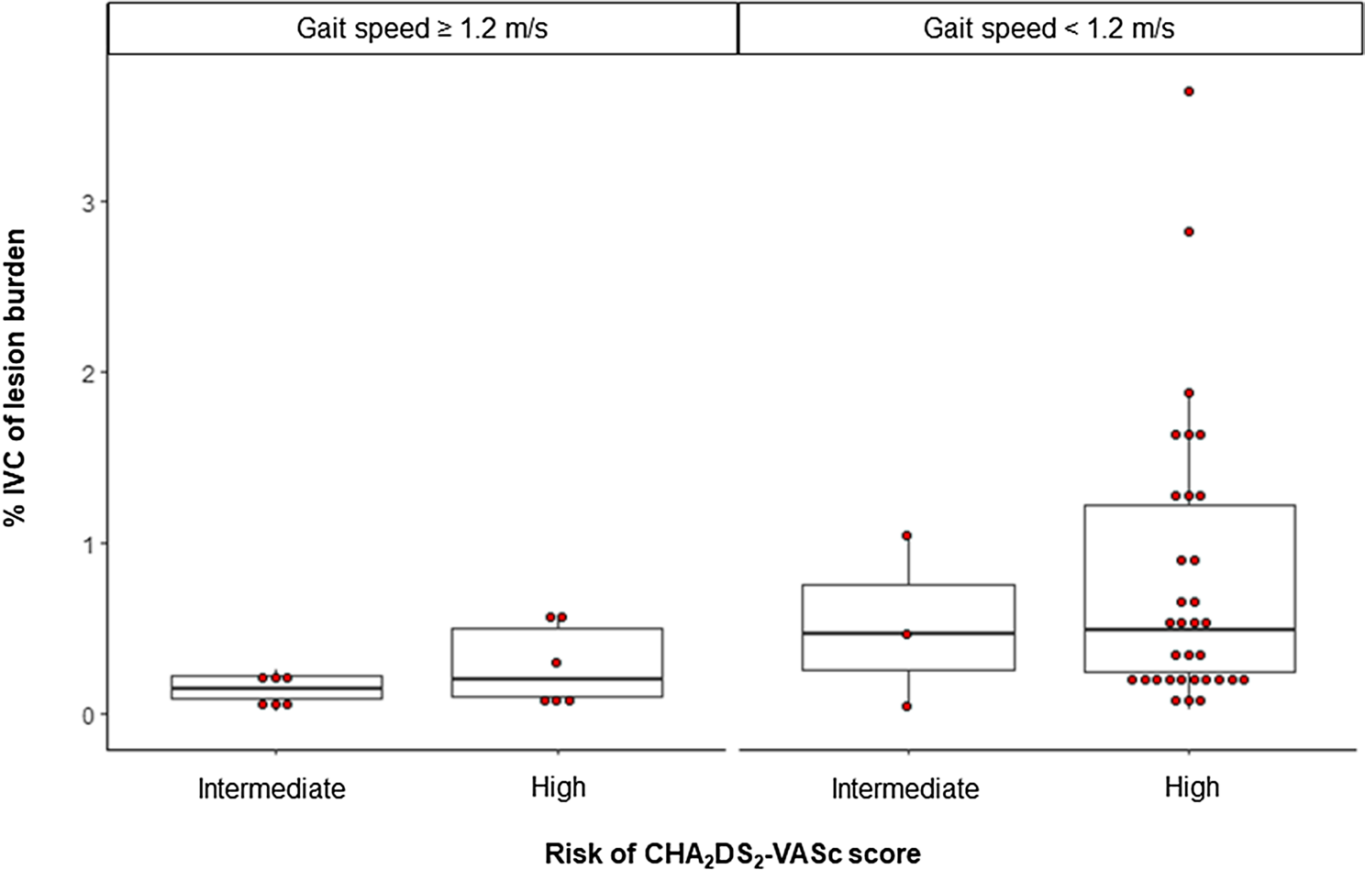
Relationship between the CHA_2_DS_2_-VASc score and brain white matter hyperintensity according to gait speed. The trend of higher white matter hyperintensity lesion burden in higher CHA_2_DS_2_-VASc score group was more prominent in slow gait speed group (right column) than the fast gait speed group (left column).

Frailty and AF are two related conditions in older individuals and are known to affect each other^21^. As one of the roles played by AF in the onset and progression of frailty, we identified that WMH lesions are highly prevalent in older adults with AF and that the WMH lesion burden is related to cardiovascular risk factors, including a higher CHA_2_DS_2_-VASc score and slow gait speed. Also, the trend of higher WMH lesion burden in higher CHA_2_DS_2_-VASc score group was more prominent in slow gait speed group (gait speed < 1.2 m/s, right column) than the fast gait speed group (gait speed ≥ 1.2 m/s, left column) (Fig. 2). Although its detailed histopathological correlates still need to be investigated, the results of this study indicate that cerebral SVD may be attributable to pathophysiology similar to that of stroke in AF patients and that cerebral SVD leads to physical frailty.

Visual assessment of WMH lesions is subject to significant interobserver variability, while volumetric measurements can provide more reliable and objective results^14,22–24^. LESIONQUANT provides accurate and reliable segmentation of multiple sclerosis lesions in contrast to manual segmentation performed by experts^25^. This automated segmentation approach also provides volumetric tracking for the identification of longitudinal trends of lesion evolution. Thus, this method can be used for further prospective longitudinal studies to assess progression of WMH lesions over time according to the CHA_2_DS_2_-VASc score or other risk factors.

Our study has some limitations. First, the findings may have limited generalisability because the study participants were relatively robust than the general older AF population owing to the exclusion of patients with symptomatic stroke and TIA and patients who could not understand the questionnaire or provided informed consent. Among them, the patients who participated in the MRI sub-study were physically stronger and functionally independent in contrast to those who were only evaluated with the CGA (Supplementary Table 3). Second, the small sample size and selection of a relatively robust population may be attributable for only the association between a lower MMSE score and small brain volume being discernible and not that with a higher WMH lesion burden. Third, we were unable to collect data on the progression of WMH lesions or occurrence of stroke, TIA, or dementia. Moreover, because of the cross-sectional design, causal relationships could not be identified. Fourth, though we recruited older AF patients and most of them (n = 110, 94%) were recommended for anticoagulation (CHA_2_DS_2_-VASc score ≥ 2), only 55.6% of the participant received anticoagulation. Difference between the cut-off value of CHA_2_DS_2_-VASc score defined in this study and recommended to anticoagulation could affect as an unmeasured confounding factor. Therefore, additional prospective study is warranted to identify correlation between anticoagulation use and WMH severity or progression in older AF patients. Despite these limitations, our results highlight the potential of SVD to be a marker or a result of aging, frailty, cardiovascular risk factors, or multimorbidity.

In conclusion, this work provides further evidence of a cross-sectional relationship between CHA_2_DS_2_-VASc scores, WMH, and frailty in older non-valvular AF patients. Cerebral SVD seemed to be more prevalent and severe among those with high risk of CHA_2_DS_2_-VASc score, which was related with slow gait speed. Further research with a longitudinal design would strengthen the evidence for the causal relationship between CHA_2_DS_2_-VASc score, cerebral SVD, and frailty in older AF population.

## Methods

### Participants

This was a prospective observational cohort study conducted at Seoul National University Bundang Hospital. Non-valvular AF patients aged ≥ 65 years were recruited from September 2015 to November 2017. The exclusion criteria were as follows: (1) heart failure in New York Heart Association classification IV, (2) unresolved malignancy, and (3) cognitive decline with inability to understand the questionnaire or provide informed consent. This prospective cohort was evaluated using three consecutive CGAs at 6-month intervals. Among the patients who participated in prospective cohort, brain MRI was additionally performed for those who consented to the sub-study. The patients who had diagnosis of symptomatic stroke or transient ischemic attack were excluded for the sub-study. Data on baseline clinicodemographic characteristics were retrieved from electronic health records (EHR).

This study was approved by the institutional review board of Seoul National University Bundang Hospital (IRB No. B-1506/304-309). Informed consent was obtained from all patients, including from those in the sub-study group, prior to enrolment. All methods were performed in accordance with relevant guidelines and regulations.

### Brain MRI protocol

MR images were acquired using 3.0-T machines (Ingenia and Ingenia CX; Philips Healthcare, Best, The Netherlands) with a 32-channel phased-array head coil. The following pulse sequences were obtained: (1) sagittal three-dimensional (3D) T1-weighted turbo field echo sequence (repetition time (TR) = 6.5 ms, echo time (TE) = 3 ms, flip angle (FA) = 9°, field of view (FOV) = 240 × 240 mm^2^, matrix = 200 × 200, slice thickness = 1.2 mm, no gap); (2) sagittal 3D T2 fluid-attenuated inversion recovery (FLAIR) sequence (TR = 4800 ms, TE = 275 ms, inversion time = 1650 ms, FA = 90°, FOV = 240 × 240 mm^2^, matrix = 240 × 240, slice thickness = 1 mm, no gap); (3) axial T2-weighted turbo spin echo sequence (TR = 3000 ms, TE = 80 ms, FA = 90°, FOV = 230 × 194 mm^2^, matrix = 420 × 300, slice thickness = 5 mm, slice gap = 1 mm); and (4) axial echo-planar diffusion-weighted image using b-values of 0 and 1000 s/mm^2^ (TR = 3000 ms, TE = 80 ms, FA = 90°, FOV = 230 × 230 mm^2^, matrix = 160 × 160, slice thickness = 5 mm, slice gap = 1 mm). The scan parameters of sagittal 3D T1-weighted imaging and sagittal 3D FLAIR imaging were based on the recommended scanner settings by NEUROQUANT and LESIONQUANT, respectively (CorTechs Labs, San Diego, CA). WMH lesions were assessed via LESIONQUANT, which offers automated quantification of WMH lesion volume, lesion burden, and counts, and anatomical lesion distribution^21^. Volume of the brain structures was measured using NEUROQUANT^26^. The burden of WMH lesions was represented with counts (number), volumes (cm^3^), % ICV, and lesion burden.

### ***CGA and CHA***_***2***_***DS***_***2***_***-VASc and HAS-BLED scores***

The CGA protocol included four domains (i.e., medical, functional, psychological, and nutrition) as previously described^27^. Comorbidity was described using the Charlson comorbidity index. Functional status was assessed using activities of daily living (ADL) and instrumental ADL (IADL). The timed up and go (TUGT) time, gait speed, and grip strength were also measured to assess physical function. Gait speed was measured using an automated laser-gated chronometer attached to the wall. Handgrip strength (kg) was measured using a Jamar Plus + Digital Hand Dynamometer (Sammons Preston, Bolingbrook, IL, USA), and the maximum value of two measurements of the dominant hand was used for the analysis. Psychological status was assessed using the Korean version of the mini-mental state examination (MMSE-KC) for cognitive function and the short form of the Korea Geriatric Depression Scale for depressive symptoms. Nutritional status was evaluated using the Mini Nutritional Assessment. We adopted cut-off values of MMSE score (≤ 24) and gait speed (< 1.2 m/s) from previous research based on the association of mild cognitive impairment and survival in older adults with the phenotype of cognitive or physical frailty^28,29^. The frailty index was calculated based on CGA^30^. ADL, IADL, TUGT time, MMSE-KC, and albumin level were used. The frailty index is a continuous variable that ranges between 0 and 1; higher scores suggest increased frailty.

The CHA_2_DS_2_-VASc and HAS-BLED scores were calculated based on the EHR. The use of anticoagulants or antiplatelet agents was also reviewed. We defined high risk CHA_2_DS_2_-VASc score as ≥ 2 in men or ≥ 3 in women and intermediate risk CHA_2_DS_2_-VASc score as 1 in men or 2 in women based on a previous study^31^. To assess baseline bleeding risk, the patients were evaluated for the HAS-BLED score, with > 3 defined as an elevated risk of bleeding^32^.

### Statistical analysis

Data were reported as means with standard deviation or medians with IQR for continuous variables and counts with percentages for categorical variables. We used the chi-square test, Fisher’s exact test, independent t-test, or Mann–Whitney U test to evaluate the associations between the CHA_2_DS_2_-VASc score, CGA variables, and brain MRI parameters. All statistical analyses were performed using SPSS version 21.0 (IBM, Armonk, NY, USA), and two-tailed *p* < 0.05 was considered significant.

## Supplementary information

Supplementary Information
