# Supplementary material for: CHA2DS2-VASc score, cerebral small vessel disease, and frailty in older patients with atrial fibrillation
Source: Sci Rep. 2020 Oct 30;10:18765. doi: 10.1038/s41598-020-75256-6 (PMC7603394; doi:10.1038/s41598-020-75256-6)
Supplement: Supplementary file 1 — Supplementary Information [file 41598_2020_75256_MOESM1_ESM.docx]

**CHA_2_DS_2_-VASc score, Cerebral Small Vessel Disease, and Frailty in Older Patients with Atrial Fibrillation**

Jung-Yeon Choi, MD, PhD^1*^; Leonard Sunwoo, MD, PhD^3,4*^; Sun-wook Kim, MD, MSc^1^; Kwang-il Kim, MD, PhD^1,2**^; Cheol-Ho Kim, MD, PhD^1,2**^

^1^Departments of Internal Medicine, Seoul National University Bundang Hospital, Seongnam, Republic of Korea; and ^2^Seoul National University College of Medicine

^3^Departments of Radiology, Seoul National University Bundang Hospital, Seongnam, Republic of Korea; and ^4^Seoul National University College of Medicine

**^*^**Jung-Yeon Choi and Leonard Sunwoo are co-first authors and equally contributed to this study.

^**^ Kwang-il Kim and Cheol-Ho Kim are co-corresponding authors.

**Correspondence to:** Kwang-il, Kim, MD, PhD,

Department of Internal Medicine, Seoul National University College of Medicine, Seoul National University Bundang Hospital, Gumi-ro 166, Bundang-gu, Seongnam-si, Kyeongi-do, 463-707, Republic of Korea; Telephone: +82-31-787-7032; Fax: +82-31-787-4052

E-mail: [kikim907@snu.ac.kr](mailto:kikim907@snu.ac.kr)

**Co-corresponding Author**

Cheol-Ho, Kim, MD, PhD,

Department of Internal Medicine, Seoul National University College of Medicine, Seoul National University Bundang Hospital, Gumi-ro 166, Bundang-gu, Seongnam-si, Kyeongi-do, 463-707, Republic of Korea; Telephone: +82-31-787-7032; Fax: +82-31-787-4052

E-mail: [cheolkim@snubh.org](mailto:cheolkim@snubh.org)

**Supplementary Figure 1. Example of periventricular (purple), deep white matter (yellow), and leukocortical (orange) white matter hyperintensities analysed using LESIONQUANT in the normal (A) and the elevated (B) CHA_2_DS_2_-VASc score group.**

Representative brain MR images of a 74-year-old man with a normal CHA_2_DS_2_-VASc score (A) and a 76-year-old woman with an elevated CHA_2_DS_2_-VASc score (B) analysed using LESIONQUANT. Periventricular, deep white matter, and leukocortical white matter hyperintensities were coloured purple, yellow, and orange, respectively.
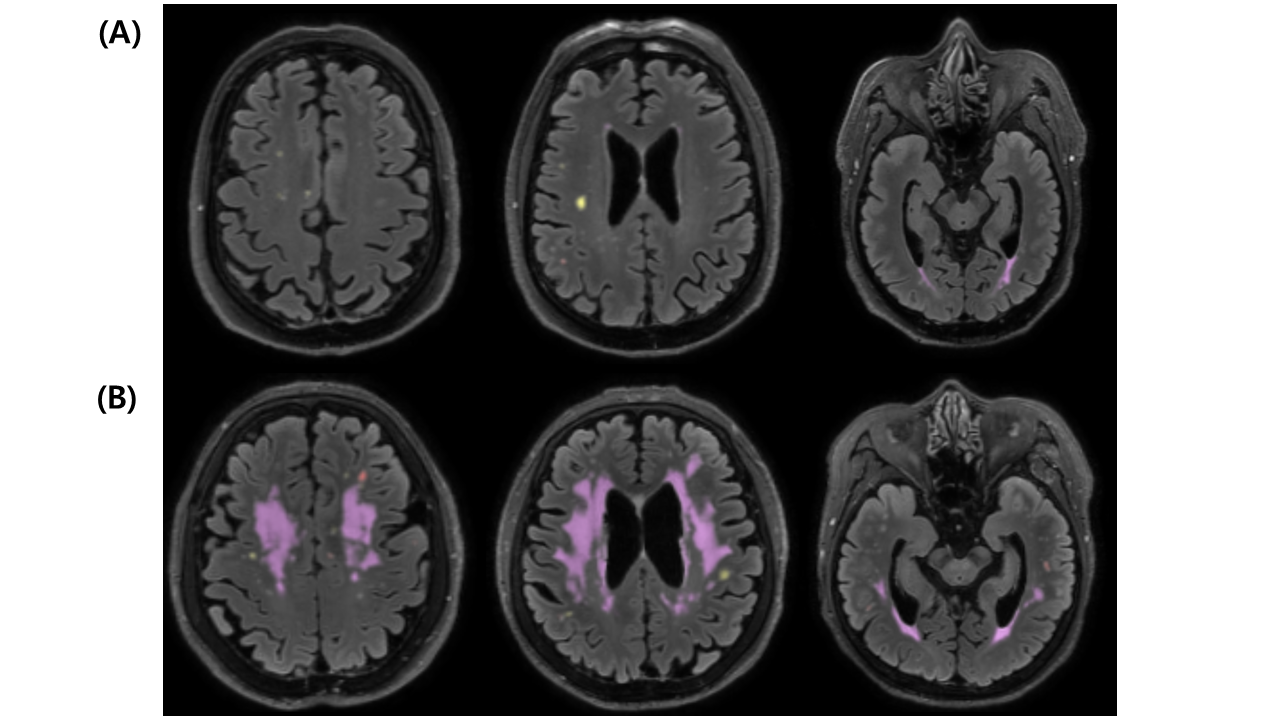


**Supplementary Table 1. Brain MRI variables according to the MMSE score**

|  | **MMSE >24**  **(n=35)** | | **MMSE ≤24**  **(n=14)** | | ***P-*Value** | |
| --- | --- | --- | --- | --- | --- | --- |
| **Brain structure** | Volume (cm^3^) | % ICV (%) | Volume (cm^3^) | % ICV (%) | volume | % ICV |
| Whole brain | 1036.9 (982.1-1090.3) | 69.0 (67.0-72.2) | 904.9 (888.8-938.4) | 69.3 (68.4-70.5) | ***<0.001*** | 0.921 |
| Superior lateral ventricles | 46.9 (30.3-61.2) | 3.08 (2.22-4.24) | 43.9 (34.3-52.1) | 3.28 (2.61-3.93) | 0.626 | 0.572 |
| Thalamus | 13.1 (12.5-14.7) | 0.90 (0.85-0.94) | 12.1 (11.4-12.7) | 0.92 (0.86-0.98) | ***0.002*** | 0.527 |
| Cortical grey matter | 449.2 (432.2-465.0) | 30.2 (28.6-31.5) | 400.7 (386.1-425.7) | 30.88 (29.55-31.74) | ***<0.001*** | 0.259 |
| Cerebral white matter | 400.2 (378.1-426.2) | 26.8 (25.6-27.9) | 332.8 (320.5-342.7) | 25.7 (24.1-26.2) | ***<0.001*** | ***0.037*** |
| 3^rd^ ventricle | 2.48 (2.12-3.18) | 0.17 (0.15-0.20) | 2.37 (2.00-2.64) | 0.18 (0.15-0.20) | 0.268 | 0.894 |
| Hippocampi | 6.23 (5.93-6.68) | 0.43 (0.38-0.45) | 5.63 (4.98-5.92) | 0.43 (0.39-0.45) | ***<0.001*** | 0.947 |
| Inferior lateral ventricles | 3.83 (2.73-5.04) | 0.25 (0.19-0.31) | 3.31 (2.78-4.04) | 0.24 (0.22-0.30) | 0.603 | 0.437 |
| **Lesion results** |  | |  | |  | |
| Lesion count (n) | 27 (16-52) | | 19 (12-32.25) | | 0.141 | |
| Lesion volume (cm^3^) | 4.11 (2.30-13.03) | | 4.44 (2.75-8.33) | | 0.833 | |
| % ICV (%) | 0.30 (0.15-0.88) | | 0.33 (0.21-0.66) | | 0.886 | |
| Lesion Burden | 1.15 (0.57-3.29) | | 1.22 (0.81-2.57) | | 0.715 | |
| **Lesion anatomical distribution (cm^3^)** | | | | | | |
| Leukocortical | 0.07 (0.01-0.37) | | 0.06 (0.00-0.34) | | 0.540 | |
| Periventricular | 3.50 (1.84-10.03) | | 4.12 (2.46-7.79) | | 0.868 | |
| Deep white matter | 0.48 (0.17-0.81) | | 0.28 (0.11-0.71) | | 0.309 | |

Data are presented as median (25–75th percentiles).

CHA_2_DS_2_-VASc, congestive heart failure, hypertension, age ≥75 (doubled), diabetes mellitus, prior stroke or transient ischemic attack (doubled), vascular disease, age 65–74, female; ICV, intracranial volume; MRI, magnetic resonance imaging**Supplementary Table 2. Brain MRI variables according to gait speed**

|  | **Gait speed ≥1.2 m/s**  **(n=12)** | | **Gait speed <1.2 m/s**  **(n=36)** | | ***P-*Value** | |
| --- | --- | --- | --- | --- | --- | --- |
| **Brain structure** | Volume (cm^3^) | % ICV (%) | Volume (cm^3^) | % ICV (%) | volume | %ICV |
| Whole brain | 1034.6 (1007.7-1106.4) | 69.5 (68.2-71.0) | 971.1 (915.7-1052.8) | 68.9 (67.2-72.0) | ***0.043*** | 0.667 |
| Superior lateral ventricles | 49.7 (31.4-72.2) | 3.28 (2.37-4.62) | 43.9 (30.1-59.6) | 3.02 (2.22-4.18) | 0.446 | 0.544 |
| Thalamus | 13.2 (12.7-15.2) | 0.90 (0.88-0.97) | 12.6 (12.1-13.8) | 0.90 (0.85-0.96) | 0.091 | 0.877 |
| Cortical grey matter | 458.1 (438.2-479.8) | 30.2 (29.4-31.9) | 432.7 (399.6-449.9) | 30.3 (28.7-31.6) | ***0.009*** | 0.868 |
| Cerebral white matter | 398.7 (381.3-439.1) | 27.0 (25.8-28.0) | 372.3 (338.2-404.8) | 26.0 (24.7-27.7) | ***0.032*** | 0.140 |
| 3^rd^ ventricle | 2.61 (2.22-3.70) | 0.19 (0.15-0.22) | 2.40 (2.10-2.84) | 0.17 (0.15-0.20) | 0.323 | 0.747 |
| Hippocampi | 6.56 (5.99-6.79) | 0.44 (0.39-0.46) | 6.02 (5.64-6.44) | 0.43 (0.38-0.45) | ***0.048*** | 0.591 |
| Inferior lateral ventricles | 3.18 (2.61-4.45) | 0.22 (0.18-0.29) | 3.79 (2.77-5.00) | 0.26 (0.22-0.33) | 0.425 | 0.095 |
| **Lesion results** |  | |  | |  | |
| Lesion count (n) | 24 (20.25-40.25) | | 24.5 (14-50.25) | | 0.934 | |
| Lesion volume (cm^3^) | 2.50 (1.19-4.05) | | 6.78 (3.03-16.38) | | ***0.012*** | |
| % ICV (%) | 0.14 (0.09-0.29) | | 0.48 (0.23-1.18) | | ***0.006*** | |
| Lesion Burden | 0.54 (0.29-1.09) | | 1.95 (0.86-4.10) | | ***0.006*** | |
| **Lesion anatomical distribution (cm^3^)** | | | | | | |
| Leukocortical | 0.03 (0.00-0.07) | | 0.12 (0.01-0.48) | | 0.052 | |
| Periventricular | 2.14 (1.04-3.46) | | 4.39 (2.45-13.28) | | ***0.011*** | |
| Deep white matter | 0.37 (0.11-0.62) | | 0.37 (0.11-1.13) | | 0.730 | |

Data are presented as median (25–75th percentiles).

CHA_2_DS_2_-VASc, congestive heart failure, hypertension, age ≥75 (doubled), diabetes mellitus, prior stroke or transient ischemic attack (doubled), vascular disease, age 65–74, female; ICV, intracranial volume; MRI, magnetic resonance imaging

**Supplementary Table 3. Clinical characteristics and comprehensive geriatric assessment according to participation in the MRI sub-study**

| **Variables** | **Substudy Group**  **(n=49)** | **Non-substudy Group**  **(n=68)** | **P value** |
| --- | --- | --- | --- |
| **Demographics** |  |  |  |
| Age (yr) | 77 (74-81.5) | 78 (75-83) | 0.153 |
| Gender, female (%) | 28 (41.2%) | 25 (51.0%) | 0.291 |
| BMI, kg/m^2^ | 24.6 (22.7-26.9) | 25.6 (23.0-27.4) | 0.485 |
| **Medication and risk stratification** | | |  |
| Antiplatelet use | 33 (48.5%) | 24 (49.0%) | 0.962 |
| Anticoagulation use | 38 (55.9%) | 27 (55.1%) | 0.933 |
| Elevated CHA2DS2-VASc^†^ | 64 (94.1%) | 40 (81.6%) | ***0.041*** |
| Elevated HAS-BLED risk (≥3) | 26 (38.2%) | 3 (6.1%) | ***<0.001*** |
| **Comprehensive Geriatric Assessment** | |  |  |
| CCI | 1 (0-2) | 2 (0.25-2) | ***0.007*** |
| ADL dependency | 13 (19.1%) | 0 (0.0%) | NA |
| IADL dependency | 18 (26.5%) | 3 (6.1%) | ***0.006*** |
| TUGT^‡^ | 11 (9.5-13) | 14 (11-16) | ***<0.001*** |
| Gait speed^§^ | 1.08 (0.89-1.21) | 0.92 (0.70-1.13) | ***0.002*** |
| Grip strength | 24.4 (19.6-32.4) | 24.6 (18.4-30.5) | 0.464 |
| MMSE-KC | 27 (23.5-28) | 25 (20.25-28) | 0.052 |
| SGDS-K^¶^ | 3 (1-5) | 3 (0.75-9) | 0.553 |
| MNA | 26 (24.25-27.25) | 25.5 (22.13-27.0) | 0.074 |
| Polypharmacy (≥ 5 drugs) | 53 (77.9%) | 33 (67.3%) | 0.200 |
| Frailty Index | 0 (0-0.06) | 0.11 (0.05-0.28) | ***<0.001*** |

Data are presented as median (25–75th percentiles) or number of participants (percentages).

^†^ Elevated CHA_2_DS_2_-VASc score refers to score ≥2 in men or ≥3 in women

^‡^ Data were missing for 12 patients.

§ Data were missing for seven patients.

^¶^ Data were missing for three patients.

ADL, activity of daily living; BMI, body mass index; CHA_2_DS_2_-VASc, congestive heart failure, hypertension, age ≥75 (doubled), diabetes mellitus, prior stroke or transient ischemic attack (doubled), vascular disease, age 65-74, female; CCI, Charlson Comorbidity Index; IADL, instrumental activity of daily living; MMSE-KC, Korean version of Mini–Mental State Examination; MNA, Mini Nutritional Assessment; SGDS-K, short form of the Korean Geriatric Depression Scale; TUGT, timed up and go test
